# Supplementary material for: Trehalose-Based Nucleolipids as Nanocarriers for Autophagy Modulation: An In Vitro Study
Source: Pharmaceutics. 2022 Apr 13;14(4):857. doi: 10.3390/pharmaceutics14040857 (PMC9026460; doi:10.3390/pharmaceutics14040857)
Supplement: Supplementary file 1 [file pharmaceutics-14-00857-s001.zip › pharmaceutics-1668089-supplementary.pdf]

Supporting Information

Trehalose-Based Nucleolipids as Nanocarriers for Autophagy Modulation: An In Vitro Study

Anthony Cunha 1,2, Alexandra Gaubert 1, Julien Verget 1, Marie-Laure Thiolat 2, Philippe Barthélémy 1, Laurent Latxague 1,†,‡ and Benjamin Dehay 2,*,‡

[1] *Univ. Bordeaux, INSERM U1212, CNRS UMR 5320, ARNA, ARN: Régulations Naturelle et Artificielle, ChemBioPharm, 146 rue Léo Saignat, 33076 Bordeaux CEDEX, France*

[2] *Univ. Bordeaux, CNRS, IMN, UMR 5293, F-33000 Bordeaux, France*

[*] *Correspondence: benjamin.dehay@u-bordeaux.fr*

† *Deceased 13 December 2021.*

[‡] *These authors contributed equally to this work.*

IR spectroscopy

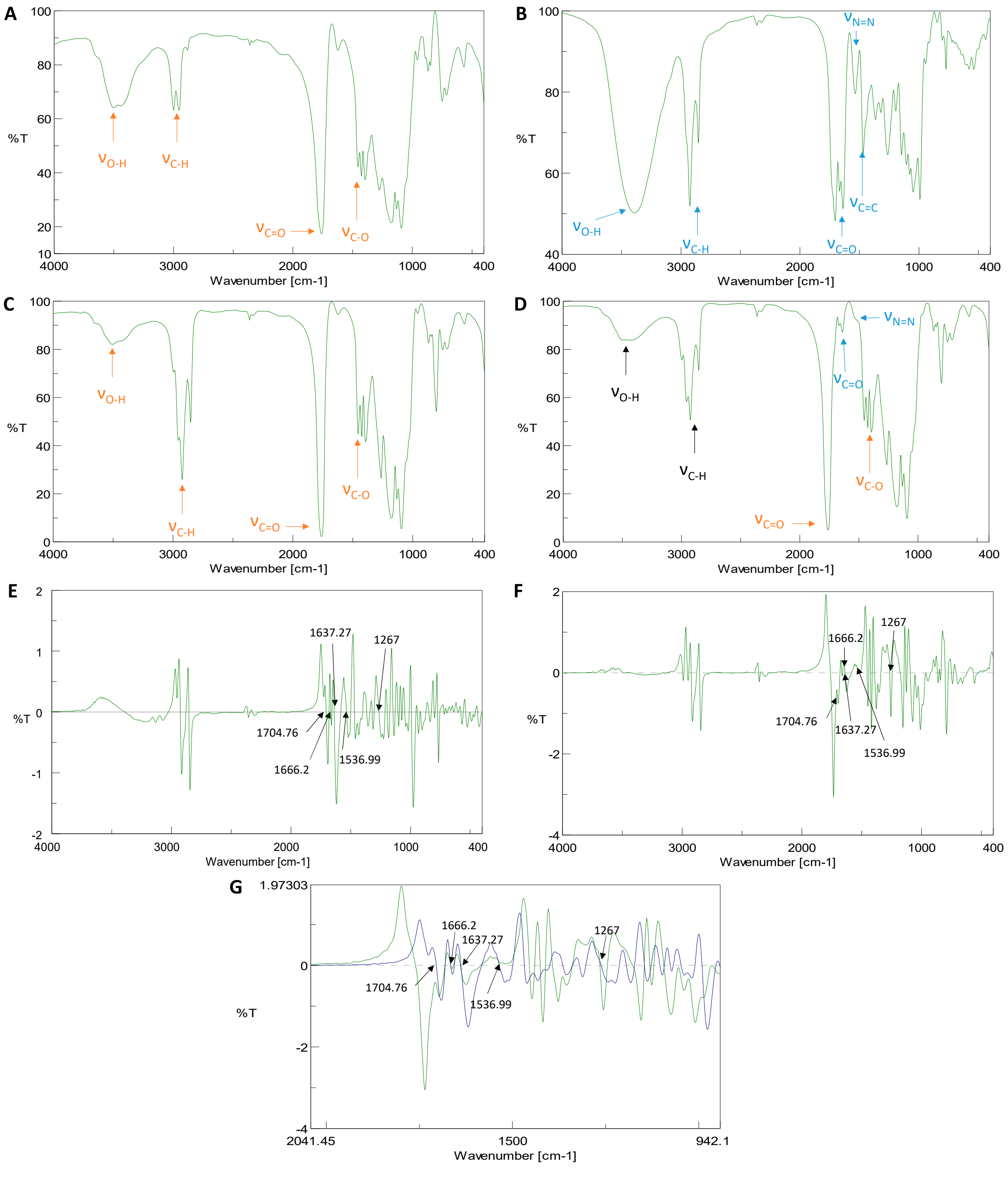

Figure S1. Infrared analysis of PLGA, GNL, and (un)loaded PLGA NPs. IR spectra of (**A**) PLGA, (**B**) GNL, (**C**) F2 and (**D**) F4, acquired in transmission mode. First derivative of (**E**) GNL, (**F**) F4 (Savitzky-Golay: Derivative order 1, Polynomic order 2 and window of 7 points) and (**G**) zoom of both first derivatives.

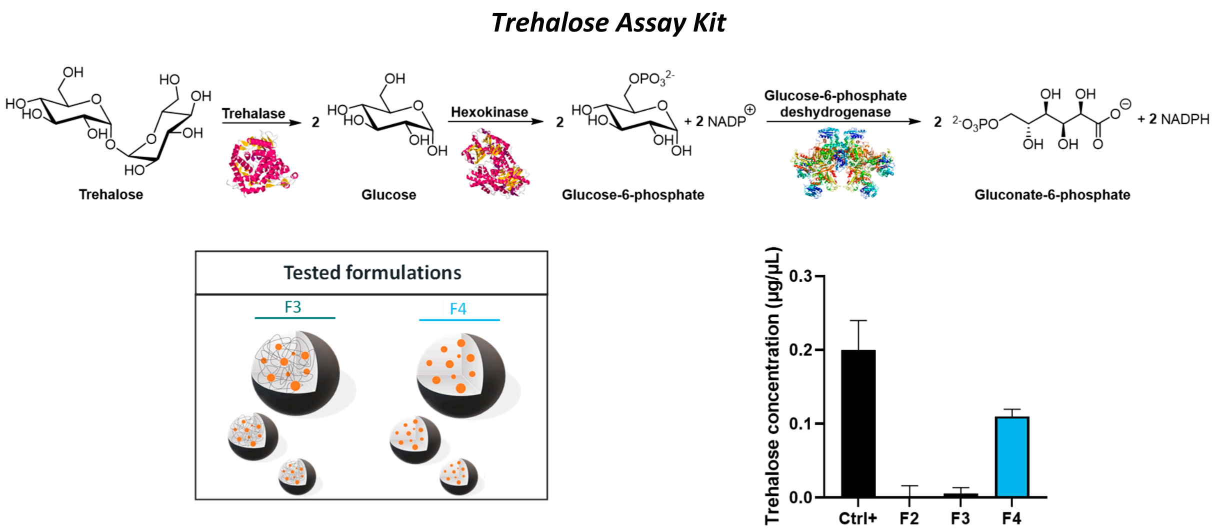

Figure S2. Principle of the enzymatic determination of trehalose. Enzymatic degradation of trehalose allowing the quantification of non-encapsulated GNL by UV spectroscopy.

UV spectroscopy

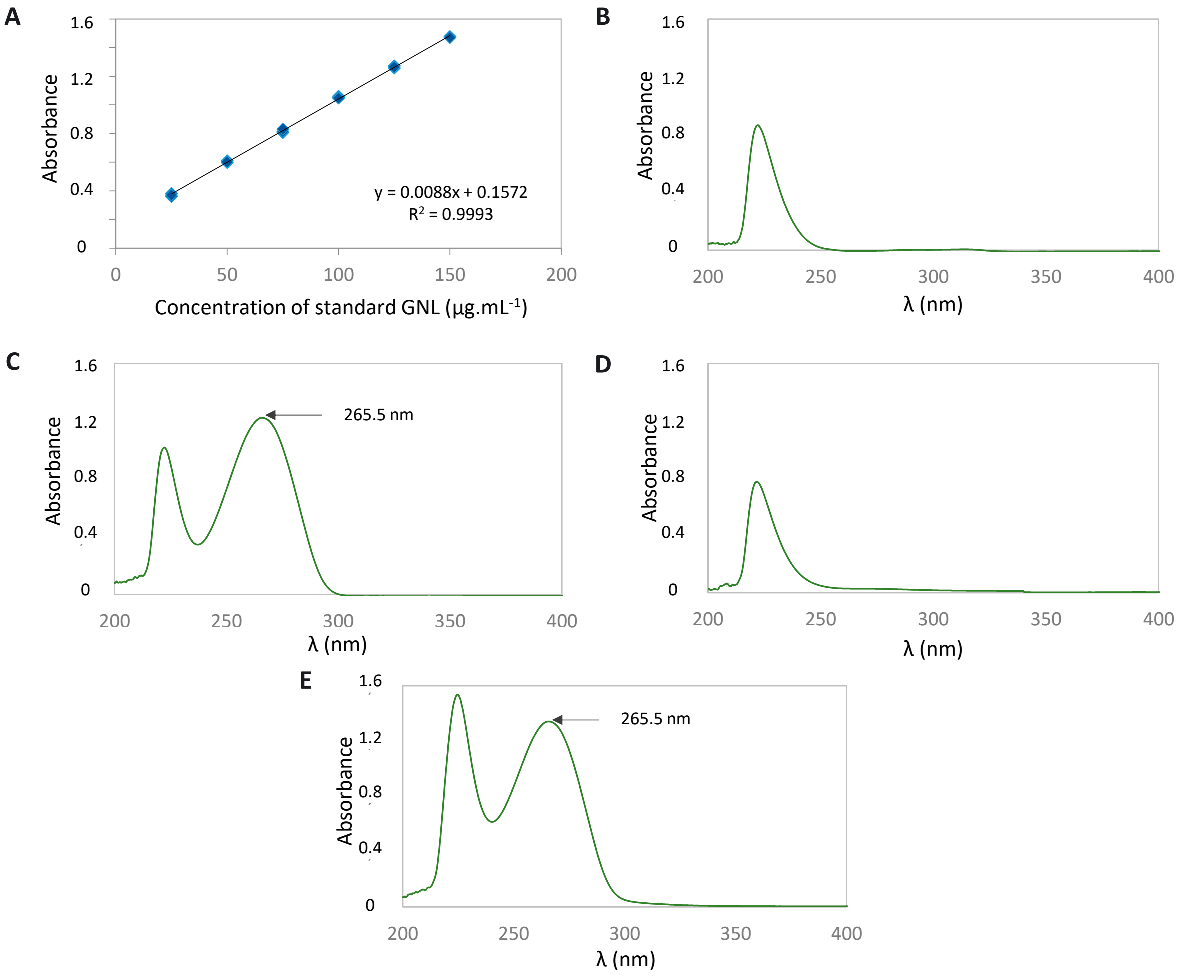

Figure S3. UV spectroscopy assays. (**A**) Calibration curve of GNL in methanol (concentration: 0.5 to 40 µg.ml^{-1}; absorbance at 266.5 nm). UV spectra of (**B**) PLGA, (**C**) GNL, (**D**) F2 and (**E**) F4.

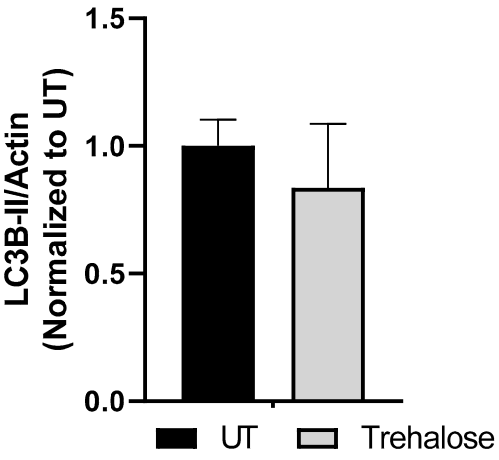

Figure S4. Biological evaluation of the condition of trehalose at the same concentration contained in the nanovectors. Quantification of LC3 protein levels normalized by actin in BE (2)-M17 cells treated with molecular trehalose for 24 h (n= 3).
